# Albuminuria Categories at Screening and Associated Clinical Factors in Adults with Type 2 Diabetes, Preserved eGFR, and No Prior Diagnosis of Diabetic Kidney Disease: An Exploratory Single-Center Cross-Sectional Study in Western Mexico

**DOI:** 10.3390/medsci14040459

**Published:** 2026-08-06

**Authors:** Martha Liliana Miranda-Martínez, Enrique Cervantes-Pérez, Sol Ramírez-Ochoa, Francisco Javier Hernández-Mora, Gabino Cervantes-Pérez, Berenice Vicente-Hernández, Alejandro González-Ojeda, Clotilde Fuentes-Orozco, Manuel Maciel-Saldierna, Enrique Rábago-Solorio, Gabino Cervantes-Guevara

**Affiliations:** 1 Departamento de Bienestar y Desarrollo Sustentable, Centro Universitario del Norte, Universidad de Guadalajara, Colotlán 46200, Mexico; dra.liliana.miranda.0212@gmail.com (M.L.M.-M.); erabagoss@hotmail.com (E.R.-S.); 2 Departamento de Medicina Interna, Hospital Civil de Guadalajara Fray Antonio Alcalde, Centro Universitario de Ciencias de la Salud, Universidad de Guadalajara, Guadalajara 44280, Mexico; enrique.cervantes@academico.udg.mx (E.C.-P.); sramirez@hcg.gob.mx (S.R.-O.); gabino-1994@hotmail.com (G.C.-P.); bvicente@hcg.gob.mx (B.V.-H.); 3 Departamento de Disciplinas Filosóficas, Metodológicas e Instrumentales, Centro Universitario de Ciencias de la Salud, Universidad de Guadalajara, Guadalajara 44340, Mexico; 4 Departamento de Clínicas, Centro Universitario de Tlajomulco, Universidad de Guadalajara, Tlajomulco de Zúñiga 45641, Mexico; 5 Departamento de Clínicas de la Reproducción Humana, Crecimiento y Desarrollo Infantil, Centro Universitario de Ciencias de la Salud, Universidad de Guadalajara, Guadalajara 44280, Mexico; frank.gine@gmail.com; 6 Departamento de Obstetricia, Hospital Civil de Guadalajara Fray Antonio Alcalde, Guadalajara 44280, Mexico; 7 Unidad Biomédica 02, Hospital de Especialidades, Centro Médico Nacional de Occidente, Guadalajara 44350, Mexico; avygail5@gmail.com (A.G.-O.); clotilde.fuentes@gmail.com (C.F.-O.); 8 Secundaria Mixta 56 “Juana de Asbaje”, Secretaria de Educación Jalisco, Guadalajara 44200, Mexico; manuel.maciel@jaliscoedu.mx; 9 Departamento de Gastroenterología, Hospital Civil de Guadalajara Fray Antonio Alcalde, Guadalajara 44280, Mexico

**Keywords:** type 2 diabetes mellitus, albuminuria, urinary albumin-to-creatinine ratio, diabetic kidney disease, glycemic control

## Abstract

Background/Objectives: Albuminuria is an important marker of kidney and cardiovascular risk in adults with type 2 diabetes mellitus (T2DM). Regional data from Mexico remain limited among patients with preserved estimated glomerular filtration rate (eGFR) and no previous diagnosis of diabetic kidney disease. This study aimed to describe albuminuria categories observed at a single screening assessment and to explore clinical factors associated with higher urinary albumin-to-creatinine ratio (uACR) categories in adults with T2DM receiving care at a regional hospital in western Mexico. Methods: This exploratory single-center cross-sectional study included 119 adults with T2DM (mean age, 53.3 ± 11.7 years), preserved eGFR (≥60 mL/min/1.73 m^2^), and no previous diagnosis of diabetic kidney disease. Albuminuria was classified from a single first-morning uACR measurement as A1 (<30 mg/g), A2 (30–300 mg/g), or A3 (>300 mg/g). Exploratory multivariable ordinal logistic regression evaluated factors associated with higher uACR categories. A secondary exploratory binary model evaluated screen-detected uACR ≥ 30 mg/g (A2/A3 versus A1). Results: A1 was observed in 67 patients (56.3%), A2 in 40 (33.6%), and A3 in 12 (10.1%); 52 patients (43.7%) had uACR ≥ 30 mg/g at screening. The median uACR was 25.6 mg/g, the median HbA1c was 11.2%, and the median fasting glucose was 280 mg/dL. In the exploratory ordinal model, longer T2DM duration (OR 1.51 per 5 years; 95% CI 1.09–2.10; *p* = 0.012), higher HbA1c (OR 1.18 per 1% increase; 95% CI 1.01–1.38; *p* = 0.036), and lower eGFR (OR 0.69 per 10 mL/min/1.73 m^2^ increase; 95% CI 0.55–0.88; *p* = 0.003) were associated with higher uACR categories after adjustment for the measured covariates. In the secondary binary model, longer T2DM duration and lower eGFR were associated with screen-detected uACR ≥ 30 mg/g. Conclusions: A single screening assessment identified uACR ≥ 30 mg/g in 43.7% of this selected regional cohort. These findings represent screen-detected albuminuria and do not establish persistent albuminuria, diabetic kidney disease, temporality, or causality. Confirmation with repeated uACR measurements and validation in larger longitudinal multicenter studies with detailed medication data are required.

## 1. Introduction

Type 2 diabetes mellitus (T2DM) is associated with microvascular and macrovascular complications, including diabetic kidney disease and cardiovascular disease [1,2,3]. Its burden remains substantial in Mexico, making early identification of kidney risk an important clinical priority [4,5,6].

Albuminuria, assessed using the urinary albumin-to-creatinine ratio (uACR), is an accessible marker of kidney damage and cardiovascular risk [7,8,9,10]. Current recommendations support periodic assessment of both eGFR and uACR in adults with T2DM [11,12]. A1, A2, and A3 categories describe increasing uACR levels, but albuminuria may vary over time; therefore, a single measurement cannot establish persistence or diabetic kidney disease [9,13,14,15,16,17,18,19,20].

Mexican studies have reported heterogeneous albuminuria estimates, likely reflecting differences in population characteristics, clinical setting, kidney function, glycemic control, and measurement strategy [21,22,23]. Local data remain limited from regional hospital populations with preserved eGFR and no previous diagnosis of diabetic kidney disease. The contribution of the present study is therefore contextual and epidemiological rather than mechanistic.

Accordingly, this exploratory single-center cross-sectional study aimed to describe screen-detected uACR categories and explore clinical factors associated with higher categories in adults with T2DM treated at a regional hospital in western Mexico.

## 2. Materials and Methods

### 2.1. Study Design and Setting

This was an exploratory single-center cross-sectional study with prospective data collection conducted at Hospital Regional de Cocula, a regional hospital in western Mexico, between September 2022 and August 2023. Recruitment was restricted to adult outpatients attending routine T2DM follow-up or a first-time evaluation. Hospitalized patients and patients seeking care for an acute intercurrent illness were not recruited. The total numbers of T2DM consultations and patients assessed for eligibility during the study period were not available.

### 2.2. Study Population

Patients were eligible if they were 20–80 years old, had T2DM, had received oral pharmacological treatment for at least 3 months, had no previous diagnosis of kidney disease or diabetic kidney disease, and had preserved kidney function (eGFR ≥ 60 mL/min/1.73 m^2^ calculated using the CKD-EPI equation). Exclusion criteria were type 1 diabetes mellitus, gestational diabetes, active urinary tract infection, fever, unusually high protein intake or protein supplementation within 24 h before sampling, nonsteroidal anti-inflammatory drug use within 24 h before enrollment, and malnutrition. Active urinary tract infection was defined as compatible urinary symptoms or clinical suspicion requiring medical evaluation or antimicrobial treatment at enrollment. Because urinary tract infection is transient, potentially eligible patients could be reconsidered after clinical resolution. Fever was defined as body temperature ≥ 38.0 °C. Malnutrition was defined according to clinical assessment documented in the medical record or evident nutritional depletion at enrollment.

#### Sample Size, Sampling Strategy, and Missing Data

Given the exploratory nature of the study, the sample size was based on feasibility and the expected number of eligible patients during the study period. Consecutive non-probability sampling was used. Eligible outpatients attending routine follow-up or first-time evaluation during the recruitment period were invited to participate until the final sample was obtained. No formal sample-size calculation for multivariable modeling was performed. Only participants with complete data for albuminuria classification and the planned analyses were included, and no missing-data imputation was performed.

### 2.3. Data Collection and Clinical Variables

Before any study procedure, written informed consent was obtained from all participants. Medical history and clinical variables were abstracted from the medical records rather than obtained through a standardized medication interview. Recorded variables included age, sex, years since T2DM diagnosis, history of hypertension, history of smoking, anthropometric measurements, blood pressure, fasting glucose, glycated hemoglobin (HbA1c), serum creatinine, lipid profile, and uACR. Body mass index (BMI) was calculated from measured weight and height, and eGFR was calculated using the CKD-EPI equation. Medication information was reviewed when available; however, complete class-level data on antihyperglycemic therapies, renin–angiotensin–aldosterone system inhibitors, sodium–glucose cotransporter 2 inhibitors, glucagon-like peptide-1 receptor agonists, and insulin were not systematically available and were therefore not included as covariates.

### 2.4. Laboratory Assessment and Albuminuria Classification

Patients were scheduled for laboratory testing after an 8–10 h fasting period. A first-morning midstream urine sample was collected for uACR measurement. Urinary albumin and creatinine were measured using an immunoturbidimetric assay on a Cobas 6000 analyzer (Roche Diagnostics, Mannheim, Germany), according to the manufacturer’s instructions and standard hospital laboratory procedures [24,25,26]. The single screening uACR value was classified as A1 (<30 mg/g), A2 (30–300 mg/g), or A3 (>300 mg/g). Because albuminuria was measured only once, these categories describe the screening assessment and do not confirm persistent albuminuria or diabetic kidney disease.

### 2.5. Outcomes

The primary outcome was the uACR category observed at the single screening assessment, classified as A1, A2, or A3. These categories represent ordered levels of the same outcome and were analyzed using exploratory ordinal logistic regression; they were not used as an independent predictor. As a secondary exploratory outcome, screen-detected uACR ≥ 30 mg/g was analyzed by combining A2 and A3 and comparing them with A1. This grouping was used for analytical simplicity and should not be interpreted as indicating that A2 and A3 confer equivalent clinical risk.

### 2.6. Statistical Analysis

Descriptive statistics were used to characterize the study population. Categorical variables are presented as frequencies and percentages. The distribution of quantitative variables was assessed using the Shapiro–Wilk test; because several variables were non-normally distributed, quantitative variables are presented as median and interquartile range (IQR). Categorical variables were compared across A1, A2, and A3 using the chi-square test or the Fisher–Freeman–Halton exact test, as appropriate. Quantitative variables were compared using the Kruskal–Wallis test, followed by Dunn’s post hoc test with adjustment for multiple comparisons when applicable. Spearman’s rank correlation coefficient was used to evaluate correlations between uACR and selected quantitative variables.

Exploratory multivariable ordinal logistic regression was performed with ordered uACR category (A1, A2, and A3) as the dependent variable. Covariates were prespecified according to clinical relevance and data availability and included age, sex, T2DM duration, history of hypertension, HbA1c, and eGFR. T2DM duration was modeled per 5-year increase and eGFR per 10 mL/min/1.73 m^2^ increase. The proportional odds assumption was evaluated using a Brant-type test; no evidence of violation was observed in the global test (*p* = 0.807). A secondary exploratory binary logistic regression used screen-detected uACR ≥ 30 mg/g (A2/A3 versus A1) as the dependent variable and included the same covariates.

Results are reported as odds ratios (ORs) with 95% confidence intervals (CIs). No formal sample-size calculation for multivariable modeling or resampling-based internal validation was performed. Given the modest sample size, six covariates, and only 12 participants in A3, estimates may be unstable and susceptible to overfitting. Accordingly, all regression analyses were considered exploratory and hypothesis-generating, and *p* values should not be interpreted as confirmatory evidence. A two-sided *p* value < 0.05 was used to identify findings warranting further investigation. Analyses were performed using SPSS Statistics for Windows, version 26.0 (IBM Corp., Armonk, NY, USA).

### 2.7. Ethical Considerations

The study was conducted in accordance with the Declaration of Helsinki and Good Clinical Practice standards [27,28]. The State Research and Ethics Committees of Jalisco approved the study (approval code: DGRPID/DI/CEI/17/22). All patients provided written informed consent before any study procedure.

## 3. Results

A total of 119 adults with T2DM met the inclusion criteria and were included in the analysis. The median age was 53 years (IQR, 45–62); 43 participants (36.1%) were men and 76 (63.9%) were women. The median T2DM duration was 10 years (IQR, 4–14), the median HbA1c was 11.2% (IQR, 9.1–13.1), and the median uACR was 25.6 mg/g (IQR, 8.3–72.8). Baseline clinical and laboratory characteristics are presented in Table 1.

At the single screening assessment, 67 patients (56.3%) were classified as A1, 40 (33.6%) as A2, and 12 (10.1%) as A3. Overall, screen-detected uACR ≥ 30 mg/g (A2/A3) was present in 52 patients (43.7%). These findings describe uACR categories at one screening assessment and do not confirm persistent albuminuria.

Categorical clinical characteristics according to uACR categories observed at screening are shown in Table 2. No statistically significant differences were observed across uACR categories for sex (*p* = 0.267), history of hypertension (*p* = 0.762), or history of smoking (*p* = 0.602).

Quantitative clinical and laboratory characteristics according to screen-detected uACR category are presented in Table 3. T2DM duration (*p* = 0.042) and HbA1c (*p* = 0.049) differed across categories in the Kruskal–Wallis analysis. Dunn’s post hoc analysis showed higher HbA1c in A3 than in A1 (adjusted *p* = 0.0481), whereas A1 versus A2 (*p* = 0.9204) and A2 versus A3 (*p* = 0.2829) were not significant. For T2DM duration, no pairwise comparison remained significant after adjustment (A1 versus A2, *p* = 0.085; A1 versus A3, *p* = 0.2559; A2 versus A3, *p* = 0.999).

Spearman correlation analysis showed weak positive correlations between uACR and T2DM duration (ρ = 0.264; *p* = 0.004) and between uACR and HbA1c (ρ = 0.290; *p* = 0.001). The correlation between uACR and eGFR was weakly negative and did not reach statistical significance (ρ = −0.163; *p* = 0.077) (Table 4).

**Figure 1:**
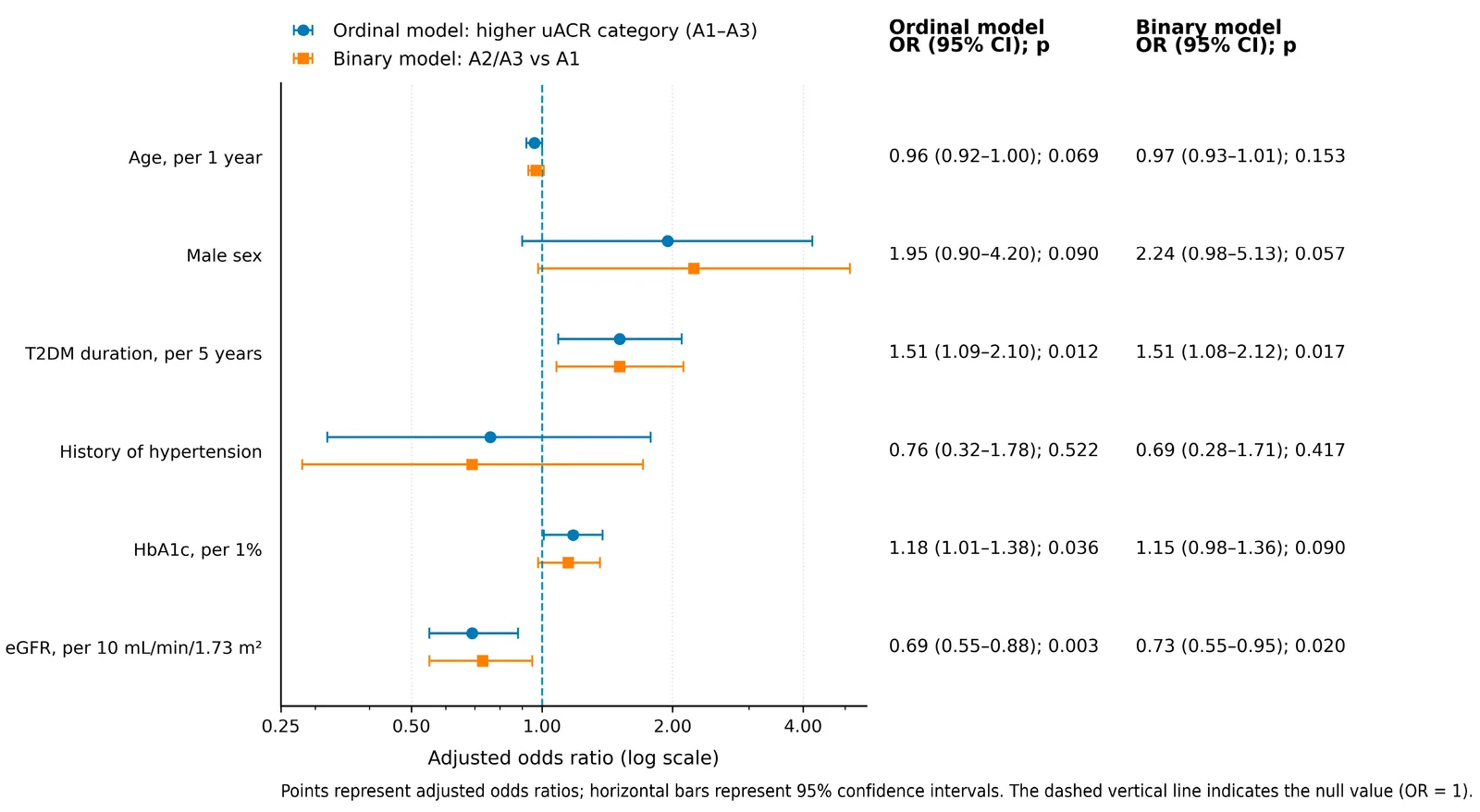
Forest plot of exploratory multivariable regression analyses of factors associated with screen-detected urinary albumin-to-creatinine ratio categories. Adjusted odds ratios and 95% confidence intervals are presented for the ordinal logistic regression model evaluating the odds of being in a higher ordered uACR category and for the binary logistic regression model comparing A2/A3 with A1. Points represent adjusted odds ratios, horizontal bars represent 95% confidence intervals, and the dashed vertical line indicates the null value (OR = 1). The horizontal axis is displayed on a logarithmic scale. Abbreviations: CI, confidence interval; eGFR, estimated glomerular filtration rate; OR, odds ratio; T2DM, type 2 diabetes mellitus; uACR, urinary albumin-to-creatinine ratio.

The results of the multivariable regression analyses are shown in Table 5. The proportional odds assumption for the ordinal logistic regression model was not violated according to the global Brant-type test (*p* = 0.807). Figure 1 shows the same results in graphical form (forest plot).

In the ordinal model, longer T2DM duration, higher HbA1c, and lower eGFR were associated with higher uACR categories after adjustment for the measured covariates. Each 5-year increase in T2DM duration was associated with higher odds of belonging to a higher uACR category (OR 1.51; 95% CI 1.09–2.10; *p* = 0.012). Each 1% increase in HbA1c was associated with higher odds (OR 1.18; 95% CI 1.01–1.38; *p* = 0.036), whereas each 10 mL/min/1.73 m^2^ increase in eGFR was associated with lower odds (OR 0.69; 95% CI 0.55–0.88; *p* = 0.003). No evidence of association was observed for age, male sex, or history of hypertension in this model. Because the analysis was exploratory and A3 included only 12 participants, the estimates should not be interpreted as robust independent predictors.

In the secondary exploratory binary model, longer T2DM duration was associated with higher odds of screen-detected uACR ≥ 30 mg/g (OR 1.51 per 5 years; 95% CI 1.08–2.12; *p* = 0.017), whereas higher eGFR was associated with lower odds (OR 0.73 per 10 mL/min/1.73 m^2^; 95% CI 0.55–0.95; *p* = 0.020). HbA1c did not reach statistical significance in the binary model (OR 1.15 per 1%; 95% CI 0.98–1.36; *p* = 0.090). The binary grouping was exploratory and should not be interpreted as indicating equivalent clinical risk in A2 and A3.

## 4. Discussion

In this exploratory single-center cross-sectional study of adults with T2DM, preserved eGFR, and no previous diagnosis of diabetic kidney disease, a single first-morning uACR assessment identified screen-detected uACR ≥ 30 mg/g in 43.7% of participants. A2 was observed in 33.6% and A3 in 10.1%. Longer T2DM duration, higher HbA1c, and lower eGFR were associated with higher uACR categories in exploratory analyses after adjustment for the measured covariates. These findings describe associations at one screening assessment and do not establish persistent albuminuria, diabetic kidney disease, temporality, progression, regression, or causality.

The frequency of A2 observed in this cohort was similar to that reported in the HOPE study and in previous Mexican cohorts [19,21,22,23]. However, direct comparisons should be made cautiously because estimates vary according to study design, clinical setting, patient selection, kidney function, glycemic control, and measurement strategy. The present cohort was recruited from a regional hospital and had markedly poor glycemic control, with a median HbA1c of 11.2% and median fasting glucose of 280 mg/dL. This characteristic may partly explain the high frequency of screen-detected albuminuria. Accordingly, the results should not be interpreted as a population-based prevalence estimate or generalized directly to all Mexican adults with T2DM or to populations with better glycemic control.

The A3 category requires particular clinical caution because uACR values were markedly elevated (median 871.1 mg/g; IQR 703.15–1426.75). Although patients with previously diagnosed kidney disease, active urinary tract infection, fever, recent NSAID use, and other potentially transient conditions were excluded, severe albuminuria may reflect previously unrecognized diabetic kidney disease, non-diabetic renal disease, or secondary and transient causes not captured by the study. The protocol did not include a complete etiologic nephrology evaluation, and neither persistence nor cause could be determined from one uACR measurement. Therefore, A3 should not be treated merely as an early screening signal or as confirmation of diabetic kidney disease. Repeated measurements and etiologic evaluation are required.

Longer T2DM duration was associated with higher uACR categories and with screen-detected uACR ≥ 30 mg/g, which is biologically plausible given the cumulative exposure to hyperglycemia and other vascular risk factors [2,3,8]. Higher HbA1c was associated with increasing uACR category in the ordinal model but not in the secondary binary model. Lower eGFR was associated with higher albuminuria categories despite all participants meeting the prespecified criterion for preserved kidney function. Albuminuria and eGFR therefore provided complementary screening information [9,11,12]. Nevertheless, the cross-sectional design cannot determine whether these factors preceded or caused the observed uACR values or whether modifying them would change albuminuria.

Sex, hypertension history, smoking history, BMI, lipid profile, blood pressure, and fasting glucose were not associated with higher uACR categories in the present analyses. These null findings should not be interpreted as evidence that the variables are clinically unimportant. The modest sample size, limited variability, possible misclassification, treatment effects, and incomplete characterization of antihypertensive and glucose-lowering therapy may have reduced the ability to detect associations. In particular, systematic class-level data on renin–angiotensin–aldosterone system inhibitors, sodium–glucose cotransporter 2 inhibitors, glucagon-like peptide-1 receptor agonists, insulin, and other therapies were unavailable. Residual confounding by treatment cannot be excluded when interpreting associations involving HbA1c, eGFR, diabetes duration, blood pressure, and albuminuria.

The secondary binary analysis combined A2 and A3 for analytical simplicity. Because these categories represent different levels of kidney and cardiovascular risk, particularly given the very high uACR values in A3, the binary model should be interpreted only as a complementary exploratory analysis and not as evidence that A2 and A3 are clinically equivalent. The ordered A1–A3 analysis remains the principal multivariable approach.

This study has several limitations. First, the single-center design, consecutive non-probability sampling, and selected regional outpatient population limit generalizability. Second, the cross-sectional design precludes assessment of temporality, causality, progression, or regression. Third, one first-morning uACR measurement cannot confirm persistent albuminuria or diabetic kidney disease because of substantial biological variability [29,30]. Fourth, the modest sample size, six-covariate models, and only 12 participants in A3 may have reduced precision and model stability and increased the risk of overfitting; no resampling-based internal validation was performed. Fifth, incomplete medication data limit control of treatment-related confounding. Sixth, the study was not designed or powered for age-stratified analyses; major cardiovascular events and relevant socioeconomic or ancestry-related variables were not systematically recorded, and eGFR estimates in older participants were not independently validated. Finally, excluding patients with previously diagnosed kidney disease allowed assessment of previously unrecognized albuminuria but may have introduced selection bias.

Despite these limitations, the study provides regional screening data from an understudied hospital-based population. Its contribution is contextual and epidemiological: documenting the frequency of screen-detected albuminuria and generating hypotheses regarding associated clinical factors rather than establishing novel mechanisms or definitive risk estimates. Larger multicenter studies with repeated uACR measurements, detailed pharmacological characterization, etiologic evaluation of severe albuminuria, socioeconomic data, and longitudinal follow-up are needed.

## 5. Conclusions

In this exploratory single-center cross-sectional study, a single first-morning uACR assessment identified screen-detected uACR ≥ 30 mg/g in 43.7% of adults with T2DM, preserved eGFR, and no previous diagnosis of diabetic kidney disease. Longer T2DM duration, higher HbA1c, and lower eGFR were associated with higher uACR categories in exploratory analyses, whereas longer T2DM duration and lower eGFR were associated with uACR ≥ 30 mg/g in the secondary binary model. Systematic uACR screening may help identify previously unrecognized elevated uACR in similar regional hospital populations. However, these findings do not confirm persistent albuminuria or diabetic kidney disease and do not establish temporality or causality. Repeated uACR measurements, detailed medication data, longitudinal follow-up, and larger multicenter cohorts are required.

## Figures and Tables

**Table 1:** Overall clinical and laboratory characteristics of the study population.

Variable	Total Cohort (*n* = 119)
Age, years	53 (45–62)
Male sex, *n* (%)	43 (36.1)
Female sex, *n* (%)	76 (63.9)
T2DM duration, years	10 (4–14)
History of hypertension, *n* (%)	48 (40.3)
History of smoking, *n* (%)	7 (5.9)
BMI, kg/m^2^	29.2 (26.2–32.7)
HbA1c, %	11.2 (9.1–13.1)
Fasting glucose, mg/dL	280 (192–369.5)
Total cholesterol, mg/dL	182.5 (151.9–224.8)
LDL cholesterol, mg/dL	113.4 (84.2–144.6)
HDL cholesterol, mg/dL	40.3 (33.0–50.7)
Triglycerides, mg/dL	220.1 (144.3–301.3)
Systolic blood pressure, mmHg	120 (111–130)
Diastolic blood pressure, mmHg	72 (70–80)
eGFR, mL/min/1.73 m^2^	105 (91.5–112)
uACR, mg/g	25.6 (8.3–72.8)
A1 (<30 mg/g), *n* (%)	67 (56.3)
A2 (30–300 mg/g), *n* (%)	40 (33.6)
A3 (>300 mg/g), *n* (%)	12 (10.1)
Screen-detected uACR ≥ 30 mg/g (A2/A3), n (%)	52 (43.7)

Values are presented as median (Q1–Q3) or *n* (%). BMI = body mass index; HbA1c = glycated hemoglobin; LDL = low-density lipoprotein; HDL = high-density lipoprotein; eGFR = estimated glomerular filtration rate; uACR = urinary albumin-to-creatinine ratio.

**Table 2:** Categorical clinical characteristics according to screen-detected uACR category.

Variable	Category	A1 (*n* = 67)	A2 (*n* = 40)	A3 (*n* = 12)	*p* Value
Sex, *n* (%)	Male	20 (29.9)	18 (45.0)	5 (41.7)	0.267
	Female	47 (70.1)	22 (55.0)	7 (58.3)	
History of hypertension, *n* (%)	Yes	27 (40.3)	15 (37.5)	6 (50.0)	0.762
	No	40 (59.7)	25 (62.5)	6 (50.0)	
History of smoking, *n* (%)	Yes	3 (4.5)	3 (7.5)	1 (8.3)	0.602
	No	64 (95.5)	37 (92.5)	11 (91.7)	

Values are *n* (% within uACR category). *p* values were calculated using the Fisher–Freeman–Halton exact test.

**Table 3:** Quantitative clinical and laboratory characteristics according to screen-detected uACR category.

Variable	A1 (*n* = 67)	A2 (*n* = 40)	A3 (*n* = 12)	*p* Value
Age, years	53 (46.5–62)	51.5 (44.8–63.2)	55 (44.8–57.2)	0.968
T2DM duration, years	8 (3–12)	11.5 (7.8–15)	13.5 (4.8–20)	0.042
BMI, kg/m^2^	30 (27.2–33.3)	28 (25.2–32.1)	29 (25.2–32.4)	0.345
HbA1c, %	10.8 (8.9–12.4)	11.2 (9.2–13.6)	12.7 (11.8–13.5)	0.049
Total cholesterol, mg/dL	183.7 (155.56–230.73)	179.27 (147.1–220.12)	189.83 (145.47–246.59)	0.794
LDL cholesterol, mg/dL	113.38 (86.19–150.06)	109.31 (84.34–130.16)	115.12 (74.66–150.32)	0.688
HDL cholesterol, mg/dL	39.6 (33.65–46.77)	42.16 (33.63–51.96)	36.93 (29.55–47.58)	0.524
Triglycerides, mg/dL	229.32 (134.97–340.59)	193.16 (135.93–261.53)	222.26 (186.17–302.37)	0.441
Systolic blood pressure, mmHg	120 (110–130)	120 (111.5–130)	128 (120–132.5)	0.251
Diastolic blood pressure, mmHg	74 (70–80)	70 (70–78.5)	73 (70–90)	0.626
eGFR, mL/min/1.73 m^2^	107 (98–112)	104.5 (90.8–110.7)	88 (65.8–110.5)	0.084
Fasting glucose, mg/dL	263 (186–335.5)	308.5 (204–422.5)	313 (227.8–379.2)	0.218
uACR, mg/g	9.1 (5.4–16.1)	69.05 (41.85–168.35)	871.1 (703.15–1426.75)	By definition

Values are median (Q1–Q3). *p* values were calculated using the Kruskal–Wallis test. Dunn’s post hoc test with adjustment for multiple comparisons was used when applicable. uACR was not compared statistically because it defines the categories.

**Table 4:** Spearman correlations between uACR and selected clinical variables.

Variable	Spearman Coefficient (ρ)	*p* Value
T2DM duration	0.264	0.004
HbA1c	0.290	0.001
eGFR	−0.163	0.077

T2DM = type 2 diabetes mellitus; HbA1c = glycated hemoglobin; eGFR = estimated glomerular filtration rate; uACR = urinary albumin-to-creatinine ratio.

**Table 5:** Exploratory multivariable regression analyses of factors associated with screen-detected uACR categories.

Variable	Ordinal Model A1–A3 OR (95% CI)	*p* Value	Binary Model A2/A3 vs. A1 OR (95% CI)	*p* Value
Age, per 1 year	0.96 (0.92–1.00)	0.069	0.97 (0.93–1.01)	0.153
Male sex	1.95 (0.90–4.20)	0.090	2.24 (0.98–5.13)	0.057
T2DM duration, per 5 years	1.51 (1.09–2.10)	0.012	1.51 (1.08–2.12)	0.017
History of hypertension	0.76 (0.32–1.78)	0.522	0.69 (0.28–1.71)	0.417
HbA1c, per 1%	1.18 (1.01–1.38)	0.036	1.15 (0.98–1.36)	0.090
eGFR, per 10 mL/min/1.73 m^2^	0.69 (0.55–0.88)	0.003	0.73 (0.55–0.95)	0.020

OR = odds ratio; CI = confidence interval; uACR = urinary albumin-to-creatinine ratio; T2DM = type 2 diabetes mellitus; HbA1c = glycated hemoglobin; eGFR = estimated glomerular filtration rate. The ordinal model evaluated ordered uACR category (A1, A2, and A3). The binary model evaluated screen-detected uACR ≥ 30 mg/g (A2/A3 versus A1). Both models included age, male sex, T2DM duration, history of hypertension, HbA1c, and eGFR. Models were exploratory because of sample-size and subgroup limitations.

## Data Availability

The data presented in this study are available on request from the corresponding author due to privacy reasons.

## Abbreviations

The following abbreviations are used in this manuscript:

A1	Normal to mildly increased albuminuria
A2	Moderately increased albuminuria
A3	Severely increased albuminuria
BMI	Body mass index
CI	Confidence interval
DKD	Diabetic kidney disease
eGFR	Estimated glomerular filtration rate
HbA1c	Glycated hemoglobin
HDL	High-density lipoprotein
IQR	Interquartile range
KDIGO	Kidney Disease: Improving Global Outcomes
LDL	Low-density lipoprotein
NSAIDs	Nonsteroidal anti-inflammatory drugs
OR	Odds ratio
SD	Standard deviation
T1DM	Type 1 diabetes mellitus
T2DM	Type 2 diabetes mellitus
uACR	Urinary albumin-to-creatinine ratio
